# Acetabular Management in Hip Hemiarthroplasty: Reaming Considerations and Current Evidence

**DOI:** 10.7759/cureus.103589

**Published:** 2026-02-14

**Authors:** Firas Hasan, Katherine Atallah, Maysaa Zahr, Ali Khalouf, Luna Maria Khalil, Alexandre El Hajj, Youssef Finianos, Mohamad Tlais

**Affiliations:** 1 Orthopedic Surgery, University of Balamand, Beirut, LBN

**Keywords:** acetabular erosion, acetabular reaming, bipolar prosthesis, hemiarthroplasty of the hip, hip fracture, revision arthroplasty

## Abstract

Acetabular reaming during hip hemiarthroplasty is practiced inconsistently across surgeons and institutions, yet its benefits and risks remain uncertain. We reviewed the clinical and biomechanical literature addressing acetabular preparation in hemiarthroplasty, including evidence related to implant seating, acetabular wear, pain, function, and later conversion to total hip arthroplasty. Across the available studies, routine reaming did not demonstrate consistent improvement in patient-reported and radiographic outcomes and may compromise cartilage and bone preservation. The decision to ream should be individualized, reserving limited, targeted reaming for cases in which acetabular anatomy prevents stable seating, while prioritizing cartilage preservation in typical fracture patients.

## Introduction and background

Femoral neck fractures are a prevalent and growing health concern in the aging population, contributing to substantial morbidity, mortality, and healthcare burden worldwide [1]. Hip hemiarthroplasty (HA) is the most commonly performed procedure for managing femoral neck fractures, particularly in elderly patients with low functional demand, as it offers several advantages over total hip arthroplasty (THA), including shorter operative time, reduced blood loss, and lower perioperative risk [2,3]. The ability to facilitate early mobilization also makes HA a practical and effective strategy for functional recovery in the elderly [1,4].

Despite its widespread use, certain technical aspects of HA remain controversial, particularly the role of acetabular reaming. Reaming involves the use of a reamer to enlarge and shape the acetabulum to accommodate the prosthetic component [5]. Proponents of reaming argue that it improves prosthetic seating, enhances biomechanical alignment, and may reduce stress on acetabular cartilage by increasing femoral head congruency [6,7]. However, critics highlight several risks, especially in osteoporotic bone, including the potential for compromised acetabular integrity, increased surgical trauma, prolonged operative time, and a heightened risk of intraoperative complications, such as mechanical injury, that could lead to accelerated cartilage erosion or necessitate earlier conversion to THA [8-10].

Although standard THA protocols emphasize acetabular reaming to ensure optimal cup orientation and fit, there is a paucity of research specifically addressing the utility and risks of reaming in hip hemiarthroplasty [6,11]. However, because HA typically articulates against retained native acetabular cartilage rather than an implanted cup, THA reaming principles are not directly transferable to HA. The existing literature on HA primarily focuses on implant type, surgical approach, or outcomes such as dislocation and acetabular erosion, but often fails to differentiate the effects of reaming [12-15]. As a result, practice variations across institutions are largely shaped by surgeon preference, implant design, bone quality, and training, rather than a robust body of comparative evidence. This highlights a significant gap in evidence-based guidance regarding the role of acetabular reaming in hip hemiarthroplasty and its potential to influence clinical outcomes and complications [8,10,16].

This review aims to systematically evaluate the available evidence on acetabular reaming in hip hemiarthroplasty. We will focus on its biomechanical rationale, clinical outcomes, and patient-specific considerations, with the goal of providing evidence-based recommendations for clinical practice.

## Review

Materials and methods

Protocol and Reporting

**Figure 1:**
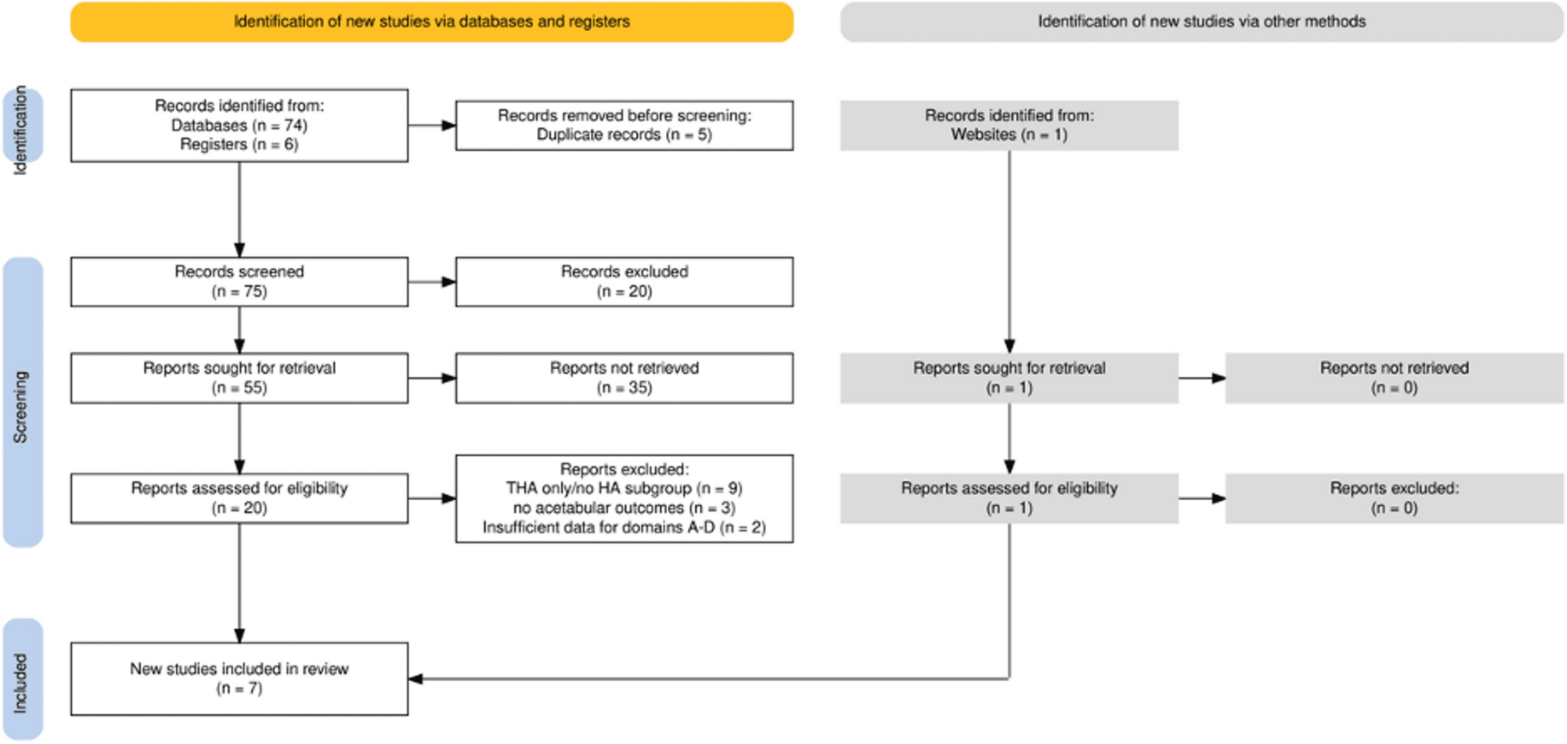
PRISMA 2020 flow diagram summarizing the study selection process for this review PRISMA: Preferred Reporting Items for Systematic Reviews and Meta-Analyses; THSA: total hip arthroplasty; HA: hemiarthroplasty

This structured review was guided by Preferred Reporting Items for Systematic Reviews and Meta-Analyses (PRISMA) 2020 principles. A formal protocol was not prospectively registered. The study selection process is summarized in the PRISMA flow diagram (Figure 1).

Search Strategy and Information Sources

A comprehensive PubMed search was performed on May 20, 2025. The following Boolean combinations were used: ("hip hemiarthroplasty" AND ("reaming" OR "acetabular erosion") AND ("unipolar" OR "bipolar") AND ("conversion to THA" OR "acetabular cartilage")). The search was limited to PubMed-indexed, English-language primary studies published from January 1, 2000, to May 20, 2025. Reference lists of eligible papers were manually screened to identify additional studies. Non-indexed materials, such as surgical technique webpages, were used solely for background information.

Eligibility Criteria

Eligible studies included adults undergoing hip hemiarthroplasty (HA) for femoral-neck or head pathology that addressed one or more of the following predefined evidence domains.

Domain A (direct): Studies that directly compare reamed vs. unreamed acetabular preparation within the same HA cohort (not inferred from implant type or other proxies).

Domain B (indirect clinical): Bipolar vs. unipolar HA as a proxy for acetabular load and erosion.

Domain C (biomechanical): Experimental analyses of reaming technique or tool accuracy.

Domain D (clinical consequence): Series or registries reporting acetabular erosion, conversion to THA, or revision risk.

The primary outcomes of interest were acetabular erosion, groin pain, function (e.g., Harris Hip Score), and conversion or revision. Conversion was defined as subsequent surgery converting HA to THA (acetabular component implantation). Revision was defined as any reoperation on the index HA construct (with or without conversion), including revision for acetabular erosion, instability, or implant failure. Randomized and non-randomized clinical trials, meta-analyses, registries, and biomechanical investigations were eligible for inclusion. Case reports, pediatric studies, and non-PubMed sources were excluded.

Study Selection and Data Extraction

Two reviewers independently screened titles, abstracts, and full texts for eligibility. Disagreements were resolved through consensus. Data extraction included study design, sample size, implant type, acetabular preparation method, follow-up duration, and outcomes. Reasons for exclusion were documented, such as studies focusing exclusively on THA or those lacking acetabular-related outcomes. At the full-text stage, we excluded articles that focused exclusively on total hip arthroplasty or that did not report acetabular-related outcomes relevant to this review (e.g., acetabular erosion/wear, groin pain, function, or conversion/revision). 

Risk-of-Bias Assessment

Risk of bias was assessed according to the study design. Because ROBINS-I (Risk of Bias in Non-randomized Studies - of Interventions) is intended for non-randomized interventional studies, it was applied only to non-randomized clinical comparative studies evaluating acetabular preparation strategies (e.g., reamed vs. unreamed within-study comparisons). Registry-based and observational cohort studies were appraised using the Newcastle-Ottawa Scale (NOS). Biomechanical/laboratory investigations were not evaluated using ROBINS-I; instead, we assessed internal validity descriptively using prespecified criteria (e.g., construct/specimen characterization, measurement methods and calibration, repeatability, and completeness of outcome reporting). Quality appraisal informed narrative weighting of evidence, and is summarized in Table 1.

**Table 1:** Risk-of-bias assessment table Overall judgment and the primary bias domains driving the rating are summarized for each included study.

Study	Risk of bias	Bias domain(s) affected	Comments
Mahmoud et al., 2024 [16]	Moderate	Confounding, outcome reporting	Retrospective design, possible confounding factors not fully controlled.
Vasileios & Spyridon, 2022 [17]	Low	No major biases identified	Literature review with solid methodological rigor.
Slotkin et al., 2017 [18]	Moderate	Measurement bias	Laboratory study; reamer wear and conditions were not perfectly controlled.
Karia et al., 2023 [5]	Low	No major biases identified	Well-controlled experimental conditions with novel reaming design.
Okike et al., 2024 [19]	Moderate	Confounding, selection bias	Registry data; lack of randomization and potential selection bias.
Bhosale et al., 2012 [20]	High	Selection bias, reporting bias	Case series; not all cases were comparable, possible reporting bias.
Reyna-Olivera et al., 2005 [21]	Low	No major biases identified	Comparative cohort study, adequate control of confounders.

Data Synthesis

Given the methodological heterogeneity and the limited number of studies directly comparing reamed vs. unreamed HA, no meta-analysis was performed. Instead, the findings were narratively synthesized across the four evidence domains to highlight consistent patterns and clinical implications. A summary table of evidence (Table 2) provides a comparison of study designs, implant types, reaming evaluation, and evidence levels.

**Table 2:** Summary evidence table Study design, implant type, whether acetabular reaming was directly evaluated, level of evidence, and the main reported outcomes (e.g., erosion, pain/function, conversion/revision) are summarized. HA: hemiarthroplasty; THA: total hip arthroplasty; MIS: minimally invasive

Study	Study design	Implant type	Reaming evaluated	Level of evidence	Key findings
Mahmoud et al., 2024 [16]	Retrospective cohort study	Unipolar/bipolar	Yes	Level III	Symptomatic acetabular erosion rare (0.48%), low conversion to THA (0.2%)
Vasileios et al., 2022 [17]	Literature review	Bipolar/unipolar	Indirect (proxy)	Level IV	Bipolar prosthesis reduces acetabular erosion compared to unipolar
Slotkin et al., 2017 [18]	Biomechanical study	N/A	Yes	Level III	Reamer accuracy depends on reamer design; MIS reamers more accurate
Karia et al., 2023 [5]	Biomechanical study	N/A	Yes	Level III	Whirlwind reaming technique more accurate than straight reaming
Okike et al., 2024 [19]	Registry-based cohort study	Unipolar/bipolar	No	Level II	Younger, healthier patients at higher risk of revision compared to THA
Bhosale et al., 2012 [20]	Retrospective case series	Unipolar	Yes	Level III	High Harris Hip Score post-THA conversion after failed hemiarthroplasty
Reyna-Olivera et al., 2005 [21]	Comparative cohort study	Unipolar/bipolar	Yes	Level II	No difference in pain/function or radiographic erosion between reamed/unreamed

Results

Overview

Seven studies met the inclusion criteria [5,16-21]. Collectively, the evidence comprised clinical comparative/observational studies [16,21], indirect clinical evidence using bipolar versus unipolar hemiarthroplasty as a surrogate for acetabular loading [17], biomechanical analyses of reaming accuracy and technique [5,18], registry-level revision data [19], and conversion outcomes following symptomatic acetabular erosion [20]. The included studies addressed one or more of four predefined domains: (A) direct clinical comparisons, (B) indirect clinical surrogates, (C) biomechanical analyses, and (D) clinical consequence data [5,16-21]. Key study characteristics are summarized in Table 1.

Synthesis of Findings Across Domains and Outcomes

Direct clinical comparisons (Domain A) provided the clearest assessment of whether acetabular reaming changes patient outcomes. In the comparative cohort evaluating reamed versus unreamed acetabular preparation, there were no significant differences in pain, functional outcomes, or radiographic acetabular erosion at mid-term follow-up [21].

Clinical consequence data (Domain D) showed that symptomatic acetabular erosion and conversion to THA were infrequent in an elderly hemiarthroplasty population in a large retrospective series [16]. In a registry-based analysis, revision risk after hemiarthroplasty compared with THA differed by patient subgroup, with higher revision risk reported in younger, healthier patients [19].

Indirect clinical surrogate evidence (Domain B) comparing bipolar and unipolar hemiarthroplasty designs reported lower acetabular erosion with bipolar implants, while functional outcomes and revision rates were not consistently different [17].

Biomechanical analyses (Domain C) reported that reaming accuracy varied with instrument wear, reamer geometry, technique, and substrate characteristics, and that certain techniques/designs improved accuracy in model systems [5,18]. Notably, much of the available biomechanical evidence relates to THA-style acetabular preparation models rather than native cartilage-bearing hemiarthroplasty articulation.

Conversion outcomes (Domain D) reported that conversion to THA after symptomatic erosion can yield favorable outcomes, although advanced erosion may require more complex reconstruction [20].

Discussion

**Figure 2:**
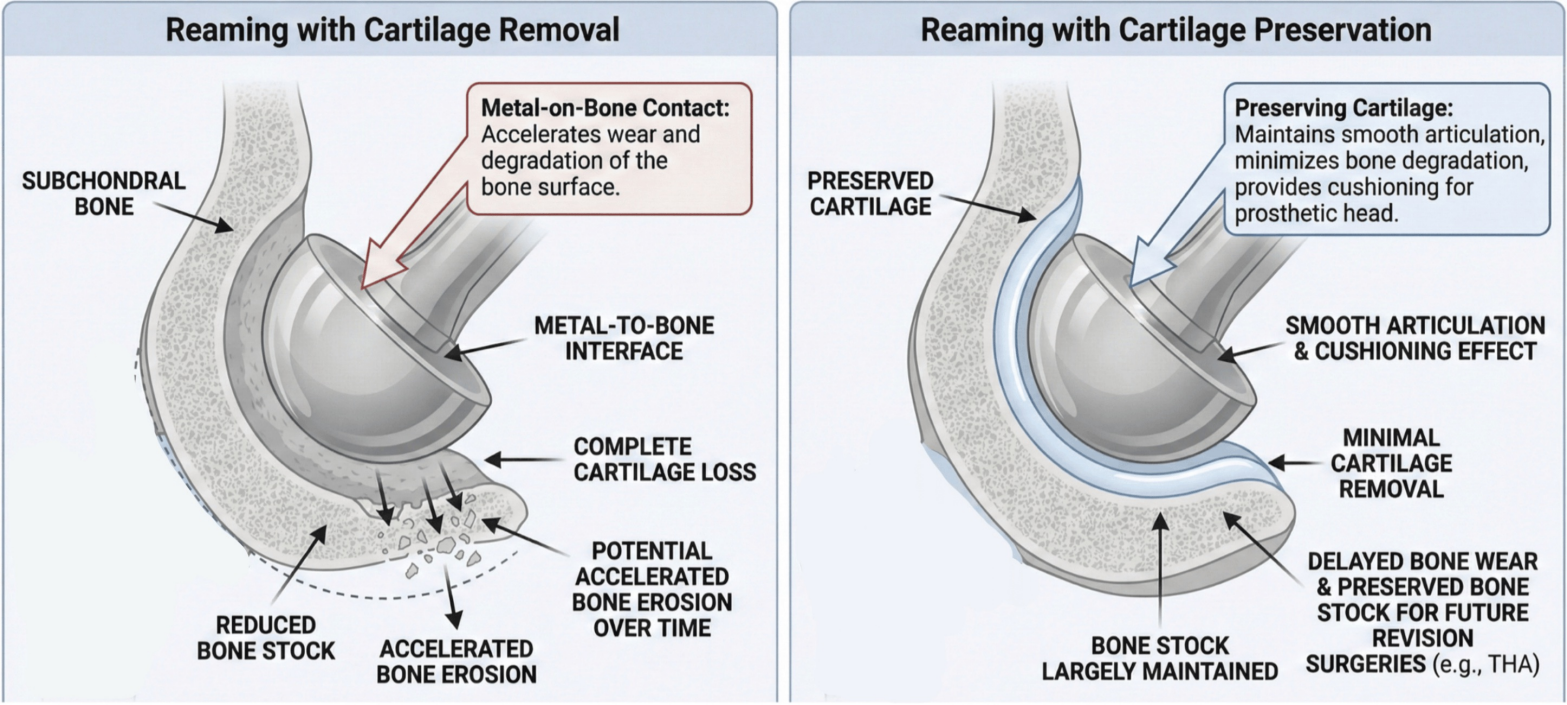
Acetabular preparation concepts in hip hemiarthroplasty Conceptual schematic comparing two approaches: (A) full cartilage removal resulting in a metal-on–subchondral bone interface, and (B) cartilage-preserving selective reaming/contouring with a predominantly metal-on-cartilage interface and preserved acetabular bone stock. THA: total hip arthroplasty Image credits: Mohamad Tlais. (Information derived from [16,21].)

The question of whether to ream the acetabulum during hip hemiarthroplasty has been longstanding and contentious. Across the limited available literature, direct clinical comparisons have not demonstrated improved pain, functional outcomes, or mid-term radiographic acetabular erosion with routine acetabular reaming during hemiarthroplasty [21]. In typical elderly fracture populations, clinically symptomatic erosion requiring conversion appears uncommon [16], whereas registry-level data suggest that revision risk after hemiarthroplasty compared with THA may be higher in younger, healthier patients [19]. Overall, the evidence is most consistent with cartilage preservation as the default approach, with selective reaming reserved for specific intraoperative indications (e.g., obstructive osteophytes, socket irregularity, or seating/congruence issues) rather than routine use [16,19,21]. Proponents of acetabular reaming argue that removing the native cartilage and creating a congruent bony socket can improve the prosthetic congruence and stability of the hemiarthroplasty. The rationale is that reaming can create a more congruent bony socket. Importantly, this biomechanical argument is largely extrapolated from total hip arthroplasty (THA) acetabular preparation literature, where reaming is performed to optimize congruence and initial acetabular component fixation. In standard hemiarthroplasty, the intended bearing is typically metal-on-cartilage, and therefore, THA-derived metal-on-bone contact/fixation principles should be interpreted cautiously and should not be assumed to translate directly to HA outcomes. When acetabular preparation is performed during HA, it is most defensible as a selective maneuver (e.g., removing obstructive osteophytes or addressing focal irregularity to improve seating) rather than routine full-cartilage removal. As illustrated in Figure 2, the impact of reaming on bone stock and cartilage preservation is significant, with full cartilage removal leading to a metal-to-bone interface, while selective minimal reaming helps preserve bone stock and cartilage, reducing potential bone erosion.

Rather than changing femoral head size, acetabular preparation may be used to improve seating and congruence of the prosthetic head within the native acetabulum when focal irregularities or osteophytes interfere with fit. Moreover, by reaming, one can remove osteophytes or irregular cartilage, eliminating impingement points and perhaps delaying localized cartilage breakdown. However, the type of reaming performed makes a significant difference: (i) Full cartilage removal is typically seen in THA-like reaming, where deeper reaming removes most, if not all, of the cartilage; (ii) Contouring of irregular osteophytes or selective minimal reaming can be beneficial in optimizing the fit without the extensive loss of cartilage. This technique focuses on smoothing areas that might cause impingement, without the need for excessive reaming that compromises the natural acetabular cartilage [18-21].

**Figure 3:**
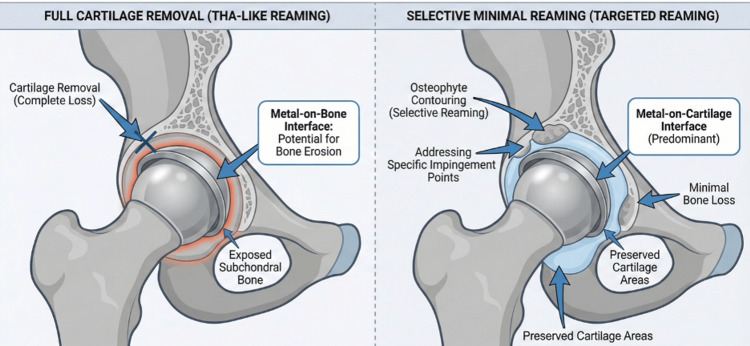
Acetabular reaming techniques in hip hemiarthroplasty Contrast between full reaming and selective, targeted reaming aimed at removing obstructive osteophytes while preserving cartilage and bone stock when feasible. Image credits: Mohamad Tlais. (Information derived from [5,16,18,21].)

Figure 3 compares the two main reaming techniques: full cartilage removal, which leads to metal-on-bone contact, and selective minimal reaming, which targets osteophyte removal while preserving most of the cartilage and minimizing bone loss.

Importantly, Reyna-Olivera et al. found no harm from reaming; at three years, reamed hips had outcomes equivalent to unreamed hips [21]. This suggests that, at least in the short term, judicious reaming does not detract from patient function or comfort, lending some support to the practice. Thus, in cases where the acetabular surface is particularly irregular or there is concern about prosthetic fit, some surgeons may lean toward limited reaming to optimize the arthroplasty biomechanics [20].

**Figure 4:**
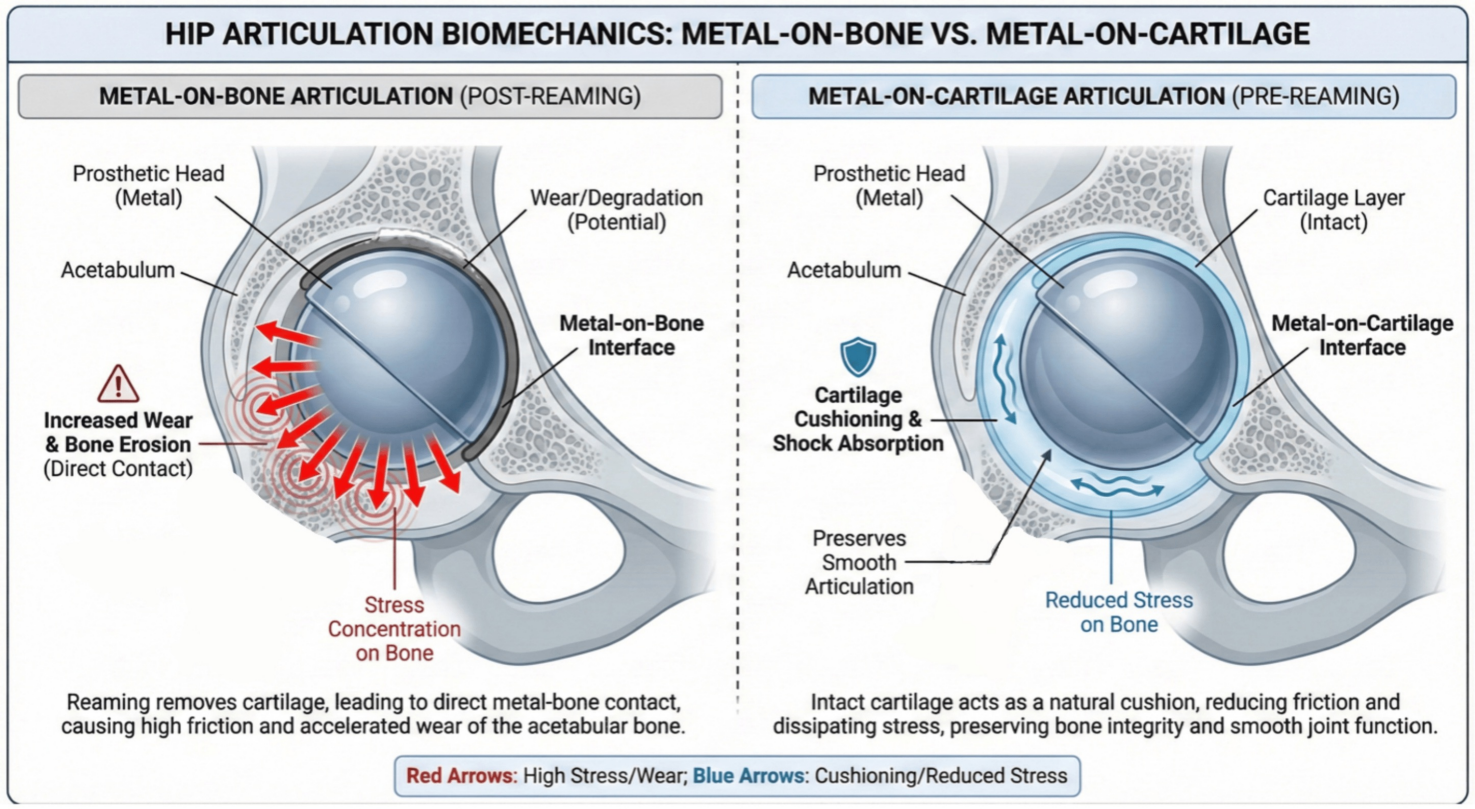
Hip articulation biomechanics; metal-on-bone vs. metal-on-cartilage Conceptual schematic contrasting direct metal-on–subchondral bone contact after cartilage removal (post-reaming) with metal-on-cartilage articulation when the cartilage layer is intact (pre-reaming). Image credits: Mohamad Tlais. (Information derived from [16,18,21].)

On the other hand, there are strong arguments against routine reaming in hemiarthroplasty. The acetabular cartilage is a natural bearing surface; removing it creates an immediate metal-on-bone articulation. This metal-to-bone contact is inherently less smooth and may accelerate acetabular wear. Essentially, reaming sacrifices the patient’s remaining cartilage “capital.” Cartilage removal can lead to faster bone erosion, potentially causing earlier onset of groin pain. Opponents point out that one of the dreaded late complications of hemiarthroplasty is acetabular protrusion - and this risk exists even without reaming, particularly in active patients (as Wheeless notes, “erosion tends to occur in active patients” with unipolar HA) [22]. If you start by removing cartilage, you may accelerate this erosion. When erosion occurs, revision is often complex (impaction grafting, sometimes a cage) [20], but conversion to THA yields good mid-term outcomes [20]. The biomechanical implications of metal-on-bone versus metal-on-cartilage articulation are shown in Figure 4, highlighting how metal-on-bone articulation can increase wear and bone erosion, while metal-on-cartilage preserves smooth joint function and reduces bone stress.

Registry data also show higher revision risk with HA vs. THA in younger, healthier patients (≈60-79; ASA I-II), with little difference in very elderly cohorts, supporting a lower threshold for primary THA in active patients [19]. While that extreme risk applies to much younger patients, it underlines the principle: younger, more active individuals will tend to erode the acetabulum if a hemiarthroplasty is in place. For an elderly, frail patient, that process might take many years or never become symptomatic; but for a fitter patient, leaving the cartilage intact at least provides an initial cushion. The data from Mahmoud et al. (0.48% symptomatic erosion overall) reinforce that in typical elderly populations, clinically important acetabular erosion is quite infrequent [16].

Thus, one could argue that routinely reaming (and thereby removing cartilage in everyone) is unnecessary “over-treatment”. Most patients will never benefit from that intervention since their cartilage will serve them well for their remaining lifetime. In summary, the case against reaming emphasizes preservation of bone and cartilage. If the acetabulum is largely intact, not reaming preserves the option of an easier conversion to THA later (since bone stock and subchondral integrity are maintained). Indeed, using a hemiarthroplasty without reaming inherently preserves acetabular bone stock, a point that may benefit patients who might require future revision [19]. Additionally, skipping reaming shortens surgical time and avoids additional bone trauma, which can be advantageous in fragile patients.

Considering these points, the clinical implications depend on patient-specific factors. In frail, elderly patients with femoral neck fracture and preserved acetabular cartilage, it is generally prudent not to ream [16]. These patients have low functional demands, and preserving cartilage maintains a smoother articulation while avoiding additional bone loss, operative time, and bleeding; available series suggest that symptomatic acetabular erosion is uncommon in this group [16].

When acetabular anatomy prevents appropriate seating of the prosthetic head (e.g., focal irregularity or large osteophytes), a surgeon may consider limited, targeted acetabular contouring/reaming. In this setting, the aim is not to ream the entire socket to bleeding bone, but to address only the focal impediment to congruence (e.g., a prominent osteophyte or high point) while preserving as much cartilage and bone stock as possible [23].

In younger or more active patients, the primary decision is often whether hemiarthroplasty is appropriate versus primary THA. If hemiarthroplasty is chosen, acetabular reaming is generally avoided to preserve acetabular cartilage and bone stock for potential future conversion, and patients should be counseled regarding the possibility of later conversion if clinically symptomatic acetabular wear develops [19].

Limitations

This review is limited by the small number of directly comparative studies; only one trial specifically contrasted reamed and unreamed hemiarthroplasty. The included evidence is heterogeneous in design, implant type, and outcome definitions, precluding meta-analysis. Restriction to English-language, PubMed-indexed studies may have excluded relevant grey or non-indexed literature. Furthermore, most clinical data are retrospective and focus on elderly fracture populations; extrapolation to younger or arthritic cohorts should be made cautiously. Future studies, particularly large-scale prospective trials, are needed to further elucidate the clinical implications of reaming and its impact on long-term outcomes.

Recommendations

Based on the synthesis of available evidence, routine acetabular reaming during hip hemiarthroplasty is not recommended for most patients. In frail elderly individuals with preserved acetabular cartilage, cartilage removal offers no functional or radiographic advantage and only increases operative time and bone loss. These patients typically achieve excellent pain relief and mobility without reaming, and the incidence of symptomatic acetabular erosion remains extremely low (≈0.5%) [16,21].

When intraoperative findings reveal focal cartilage irregularities or obstructive osteophytes that prevent proper seating of the prosthetic head, a limited and selective reaming approach can be considered. The goal should be to remove only high points or deformities while preserving as much cartilage as possible, thereby improving congruence without creating a full metal-on-bone interface [5,23].

For younger or more active patients (typically under 80 years or with ASA I-II status), the long-term durability of a hemiarthroplasty is inferior to that of THA. In such cases, if a hemiarthroplasty is chosen, acetabular reaming should be avoided to preserve bone stock and delay the onset of cartilage wear. A bipolar prosthesis may be preferred over a unipolar design, as it reduces contact stress on the acetabular surface and delays erosion [17,19].

In patients with poor bone quality or osteoporotic acetabula, aggressive reaming should also be avoided due to the heightened risk of cortical compromise and intraoperative fracture [8-10]. When reaming is technically required (such as for irregular sockets or in cases of congenital or post-traumatic deformity), surgeons should use sharp, well-calibrated reamers. Evidence suggests that worn or conventional hemispherical reamers tend to under-ream by about 1.3 mm, whereas newer minimally invasive or “whirlwind” reamer designs achieve superior accuracy and surface conformity [5,18].

Overall, the literature supports a selective, patient-specific approach to acetabular reaming: cartilage preservation should remain the default strategy, and reaming should be reserved only for anatomical or technical situations where its benefits clearly outweigh its risks. Primary THA should be considered first in younger, healthier, and more active patients, as it addresses acetabular pathology directly and may reduce the need for later conversion compared with hemiarthroplasty. In such patients, recommending acetabular reaming during hemiarthroplasty is not a substitute for THA; rather, selective minimal acetabular contouring (if used at all) is most applicable when hemiarthroplasty is performed for specific reasons (e.g., patient preference, limited resources, or contraindications to THA) and when focal acetabular irregularity or osteophytes prevent appropriate seating of the prosthetic head.

## Conclusions

In conclusion, our evidence-based review indicates that routine acetabular reaming is not recommended for most hip hemiarthroplasty cases. There is no evidence of improved pain or function from routine reaming in the short term. In typical elderly hemiarthroplasty cohorts, the reported incidence of symptomatic acetabular erosion requiring conversion is low (e.g., 0.48% in a large retrospective series). The default approach should be to preserve the acetabular cartilage. Reaming should be considered only in select situations where clear benefits outweigh the downsides, for example, removing an impinging osteophyte or achieving a stable reduction in a mildly deformed acetabulum. Adopting a patient-specific strategy (as outlined above) aligns with the literature: use HA (without reaming) for low-demand patients, and reserve more invasive measures (reaming or even primary THA) for those who are biologically young or have pre-existing acetabular issues. This tailored approach maximizes each patient’s outcome while minimizing unnecessary acetabular damage. Ultimately, ongoing follow-up and additional high-quality studies (particularly randomized trials) will help refine these recommendations, but current evidence supports a selective, case-by-case decision on reaming rather than a one-size-fits-all rule.

## References

[1] Ho JP, Wong AY, Ong LH (2023). Mobility and hip function among geriatric patients with displaced neck of femur fractures treated with arthroplasty. Geriatr Orthop Surg Rehabil.

[2] Sekeitto AR, Sikhauli N, van der Jagt DR, Mokete L, Pietrzak JR (2021). The management of displaced femoral neck fractures: a narrative review. EFORT Open Rev.

[3] Zeng Z, Li H, Luo C (2025). Risk factors associated with mortality in elderly patients receiving hemiarthroplasty for femoral neck fractures. BMC Musculoskelet Disord.

[4] Bayar E, Cengiz T, Aydın Şimşek Ş, Albayrak B, Büyükceran İ, Tomak Y (2025). Managing mortality: key factors influencing hemiarthroplasty outcomes in geriatric patients with proximal femur fractures. Medicina (Kaunas).

[5] Karia M, Boughton O, Mohan SV, Halewood C, Wozencroft R, Clarke S, Cobb J (2023). Enhancing acetabular reaming accuracy: optimal techniques and a novel reamer design. J Orthop Surg Res.

[6] Zuo J, Xu M, Zhao X, Shen X, Gao Z, Xiao J (2021). Effects of the depth of the acetabular component during simulated acetabulum reaming in total hip arthroplasty. Sci Rep.

[7] Hickernell TR, Kaidi AC, Davignon R, Geller JA, Cooper HJ, Shah RP (2020). Deeper central reaming may enhance initial acetabular shell fixation. Arthroplast Today.

[8] Gautreaux M, Kautz S, Martin Z (2023). Acetabular wall weakening in total hip arthroplasty: a pilot study. Pathophysiology.

[9] Beckers G, Djebara AE, Gauthier M (2022). Acetabular peri-prosthetic fractures-a narrative review. Medicina (Kaunas).

[10] Kusuma S, Goodman Z, Sheridan KC, Wasielewski R (2013). The effects of acetabular reaming on bone loss and component coverage. Orthop Proc.

[11] Tanaka T, Kaneko T, Hidaka R, Hashikura K, Ishikura H, Moro T, Tanaka S (2020). Midterm results of revision total hip arthroplasty for migrated bipolar hemiarthroplasty in patients with hip osteoarthritis using cementless cup with the rim-fit technique. J Orthop Surg (Hong Kong).

[12] Robertson GA, Wood AM (2018). Hip hemi-arthroplasty for neck of femur fracture: what is the current evidence?. World J Orthop.

[13] Gezahegn B (2023). Hemiarthroplasty. Arthroplasty - Advanced Techniques and Future Perspectives.

[14] Bush JB, Wilson MR (2007). Dislocation after hip hemiarthroplasty: anterior versus posterior capsular approach. Orthopedics.

[15] Leonardsson O, Kärrholm J, Åkesson K, Garellick G, Rogmark C (2012). Higher risk of reoperation for bipolar and uncemented hemiarthroplasty. Acta Orthop.

[16] Mahmoud AN, Suk M, Horwitz DS (2024). Symptomatic acetabular erosion after hip hemiarthroplasty: is it a major concern? A retrospective analysis of 2477 hemiarthroplasty cases. J Clin Med.

[17] Vasileios A, Spyridon P (2022). Dissociation of bipolar hemiarthroplasty of the hip and review of literature. Arthroplast Today.

[18] Slotkin S, Frisch NB, Roc G, Silverton CD (2017). Hemispherical and minimally invasive total hip reamers: a biomechanical analysis of use and design. Arthroplast Today.

[19] Okike K, Prentice HA, Chan PH (2024). Unipolar hemiarthroplasty, bipolar hemiarthroplasty, or total hip arthroplasty for hip fracture in older individuals. J Bone Joint Surg Am.

[20] Bhosale P, Suryawanshi A, Mittal A (2012). Total hip arthroplasty for failed aseptic Austin Moore prosthesis. Indian J Orthop.

[21] Reyna-Olivera G, Harb-Peña EJ (2005). Clinical and X-ray comparison between a reamed versus an unreamed acetabulum in hip hemiarthroplasty. Acta Ortop.

[22] Wheeless CR (2026). Hemiarthroplasty of the hip. Wheeless’ Textbook of Orthopaedics. Published July 22.

[23] Karelse A, Leuridan S, Van Tongel A, Debeer P, Van Der Sloten J, Denis K, De Wilde LF (2015). Consequences of reaming with flat and convex reamers for bone volume and surface area of the glenoid; a basic science study. J Orthop Surg Res.

